# Micro-Viscometer for Measuring Shear-Varying Blood Viscosity over a Wide-Ranging Shear Rate

**DOI:** 10.3390/s17061442

**Published:** 2017-06-20

**Authors:** Byung Jun Kim, Seung Yeob Lee, Solkeun Jee, Arslan Atajanov, Sung Yang

**Affiliations:** 1 Department of Biomedical Science and Engineering, Gwangju Institute of Science and Technology, Gwangju 61005, Korea; gene392@gist.ac.kr (B.J.K.); syl@gist.ac.kr (S.Y.L.); 2 School of Mechanical Engineering, Gwangju Institute of Science and Technology, Gwangju 61005, Korea; sjee@gist.ac.kr (S.J.); arslanrcx@gist.ac.kr (A.A.)

**Keywords:** micro-viscometer, shear-varying viscosity, blood viscosity, microchannel array

## Abstract

In this study, a micro-viscometer is developed for measuring shear-varying blood viscosity over a wide-ranging shear rate. The micro-viscometer consists of 10 microfluidic channel arrays, each of which has a different micro-channel width. The proposed design enables the retrieval of 10 different shear rates from a single flow rate, thereby enabling the measurement of shear-varying blood viscosity with a fixed flow rate condition. For this purpose, an optimal design that guarantees accurate viscosity measurement is selected from a parametric study. The functionality of the micro-viscometer is verified by both numerical and experimental studies. The proposed micro-viscometer shows 6.8% (numerical) and 5.3% (experimental) in relative error when compared to the result from a standard rotational viscometer. Moreover, a reliability test is performed by repeated measurement (N = 7), and the result shows 2.69 ± 2.19% for the mean relative error. Accurate viscosity measurements are performed on blood samples with variations in the hematocrit (35%, 45%, and 55%), which significantly influences blood viscosity. Since the blood viscosity correlated with various physical parameters of the blood, the micro-viscometer is anticipated to be a significant advancement for realization of blood on a chip.

## 1. Introduction

Blood provides the most diverse and complex source of information about a body among human biofluids. Decades of studies have shown that the physical parameters of blood correlate with various cardiovascular diseases, such as hypertension [1,2], diabetes [3], myocardial infarction [4], and coronary heart disease [5]. Therefore, it is expected that a comprehensive analysis of various physical characteristics of blood can enable an accurate and rapid diagnosis of cardiovascular diseases or aid in patient monitoring. Specifically, blood viscosity is known as a representative indicator that is influenced by blood cell characteristics (such as hematocrit, deformability, aggregation) and plasma viscosity [6]. Thus, the accurate measurement of blood viscosity is essential for a comprehensive analysis of the various physical indicators in blood. 

Viscosity as a hydrodynamic term is a measure of the resisting force acting against the applied shear force. Blood, especially, is a representative non-Newtonian fluid, i.e., its viscosity varies according to the shear rate. This implies that the most important objective in measuring blood viscosity is to obtain not only a single viscosity value at a specific shear rate, but also multiple viscosity values under a wide range of shear rates.

Conventional methods for measuring viscosity on a macroscopic scale include respective use of a capillary viscometer, falling ball viscometer, and rotational viscometer [7]. By leveraging gravitational force, methods of measuring the speed of a capillary-driven fluid or the velocity of a ball falling in a fluid-filled tube have been developed. These classic methods are accurate; nevertheless, they have limitations in actively controlling shear rate conditions. Rotational viscometers that have either a coaxial cylinder or a cone and plate overcome this problem by varying the angular velocity of the cylinder or cone. It is additionally possible that a wide shear rate condition may be required to measure the viscosity of a non-Newtonian fluid such as blood. However, this approach is disadvantageous in terms of the requisite bulky and expensive equipment, large sample consumption, and long measurement time.

The above limitations have led to the introduction of microfluidic viscosity measurement techniques that can measure viscosity within a short period of time with only a small amount of sample. Some of the microfluidic viscometers operate with special systems (e.g., electro wetting on dielectric [8], an optical tweezer [9], and a magnetic field [10]). However, owing to the complexity of sensors and operating systems, capillary-driven and pressure-driven viscometers have been widely studied.

The capillary-driven viscometer can estimate viscosity by measuring the velocity of a sample fluid flowing inside the capillary channel with a serpentine configuration [11,12]. In addition, a viscometer that can measure non-Newtonian fluidic viscosity by measuring the average velocity of a sample fluid decelerating in a capillary channel has been reported [13]. The device employing this concept is easy to fabricate, and the viscosity measurement is possible with an extremely small amount of sample fluid. However, a capillary flow is very sensitive to surface properties and thus requires very similar surface states for each device in order to guarantee reliable measurement performances. Furthermore, within a given geometry, the measurable shear rate range cannot be actively manipulated.

In contrast to capillary-driven methods, many pressure-driven viscometers that are capable of active shear rate control have been developed. Various approaches exist for measuring the viscosity by means of measuring various mechanical and electrical factors, such as the vibrational noise spectrum [14], ring-down voltage from the vibrating wire [15], voltage drop by cantilever deflection [16], and electrical resistance of the blood flow [17]. These methods enable an accurate viscosity measurement with a very small amount of sample fluid; nonetheless, they require complex devices and systems. Meanwhile, a method for estimating viscosity by measuring pressure in a micro-channel was developed [18,19]. Although it does not require a complicated device configuration, electrodes, as well as a calibration procedure that converts pressure into viscosity, is necessary. For this reason, the method of analyzing the fluidic boundary by using the reference fluid with the known viscosity has the advantage of not requiring an additional electrode. Based on this idea, methods of measuring viscosity by image acquisition and processing of the cross-sectional area [20] or channel width [21] at a given flow rate were developed. Meanwhile, another device measures viscosity using the values of a flow rate ratio at a given boundary position [22]. Another group proposed a method of determining a flow rate ratio at the moment of a reverse flow occurring on account of the hydrodynamic balancing in a junction channel [23,24]. Our group developed a viscosity measurement technique using hydraulic compartments [25,26], which can measure the viscosity by simply counting the number of channels filled with reference and sample fluids. The above methodologies do not require an electrode or complicated device configurations. Nevertheless, they provide a single viscosity value at a particular shear rate from a given flow rate condition. For the case of viscosity measurements of a non-Newtonian fluid, such as blood, multiple shear rate conditions with respect to the number of viscosity data are crucial to obtain.

Herein, a pressure-driven micro-viscometer that can acquire a shear-varying blood viscosity over a wide range of shear rates is proposed. Unlike previous pressure-driven viscometers, the main feature of the proposed viscometer includes multiple shear rate generation at a fixed flow rate. This is realized by an optimal design of the multiple microchannel arrays in the viscometer. Since it obtains 10-fold more data points of viscosity at a given flow rate condition, this feature is highly beneficial, especially for measuring the shear-varying viscosity of a non-Newtonian fluid like blood. The functionality of this viscometer is demonstrated by experimental demonstration as well as numerical simulation, and is compared to a standard rotational viscometer.

## 2. Materials and Methods 

### 2.1. Numerical Simulation

A numerical simulation was performed using COMSOL Multiphysics (COMSOL Inc., Burlington, MA, USA) to verify the performance of the proposed micro-viscometer. In this simulation, the incompressible laminar flow was assumed because it is a microscale phenomenon. The Laminar Two-Phase Flow—Phase Field module was exploited in order to simulate the Newtonian (reference) and non-Newtonian (sample) fluids flowing together in the viscometer. The boundary condition for the inlet was set to mass flow since the fluids are infused at a specific flow rate by a syringe pump. The outlet of the device was in an open state, thus it was set to zero pressure. Also, the channel walls had a no-slip condition. The properties and conditions of the reference and sample fluids used in the simulation are summarized in Table 1. It is notable that the sample fluid viscosity was expressed by the power law model, which is a general method for describing a shear-varying viscosity by using two parameters (flow consistency index k, and flow behavior index n). These parameters were acquired for a comparison with the result from the viscosity measurements by using a rotational viscometer known to be the “gold standard” for measuring viscosity. As an initial condition, the reference and sample fluids were symmetrically filled in their respective halves of the device according to the initial interface located at the center of the device. For the time-dependent solution, PARDISO solver was utilized from 0 to 1000 s with a time interval of 10 s. The tolerance was set to be 5 × 10^−4^.

### 2.2. Fabrication of the Micro-Viscometer

The micro-viscometer mold was fabricated using a conventional photolithography technique by spin-coating negative photoresist (SU-8 2025, MicroChem, Westborough, MA, USA) with the height of 50 μm on a silicon wafer. A polydimethylsiloxane (PDMS; Sylgard 184 A/B, Dow Corning, Seoul, Korea) replica was prepared from the SU-8 mold using a typical soft lithography technique. From that point, the PDMS replica and glass slide were irreversibly bonded via oxygen plasma treatment (COVANCE, Femto Science, Yongin, Korea). The fabricated viscometer had two inlets for injection of the reference and sample fluids, as well as an open-state outlet.

### 2.3. Experimental Setup

The rotational viscometer (HAAKE MARS II, Thermo Scientific, Waltham, MA, USA), which is known to be a gold standard for viscosity measurements, was used to obtain the reference data for evaluating the measurement accuracy of results from both the numerical and experimental analyses. Twenty sets of viscosity in the shear rate condition of 10–1000/s were obtained from the rotational viscometer. The two indices from the power-law model (k and n) were acquired using regression analysis. 

Newtonian and non-Newtonian fluids were used for the experimental demonstration. For the Newtonian fluid, 1% (*v*/*v*) fluorescent particle (0.3 μm in diameter) suspension in DI water was used as a reference fluid and DI water mixed with 8% Sodium Dodecyl Sulphate (SDS) was used as a sample fluid. For the non-Newtonian fluid, Phosphate Buffered Saline (PBS; pH 7.4, Gibco, Invitrogen, Carlsbad, CA, USA) was used as a reference fluid and the red blood cell suspension in PBS was used as a sample fluid. The packed red blood cells employed for the sample fluid were supplied by Gwangju Chonnam blood bank branch of the Korean Red Cross for research purposes only. Its use was approved by the Institutional Review Board at Gwangju Institute of Science and Technology (20160328-BR-22-03-31). In the sample preparation step, the packed red blood cells obtained from the blood bank were washed by mixing with PBS and centrifuging at 3500 RPM for 5 min. After centrifugation, PBS and supernatant cells were discarded. This consecutive step was conducted twice in order to completely remove the media containing the packed red blood cells. Finally, the remaining red blood cells were mixed with PBS. The volume of the red blood cells and PBS varied according to the hematocrit. 

In the experiment, a microscope (BX51, Olympus, Tokyo, Japan) equipped with a charge-coupled device (CCD) camera (DP72, Olympus, Tokyo, Japan) and a 1.25x objective lens (MPLFN 1.25X, Olympus, Tokyo, Japan) was used to count the number of channels filled with each fluid. Two syringe pump modules (neMESYS, CETONI GmbH, Korbußen, Germany) were used to inject the reference and sample fluids.

### 2.4. Viscosity Measurement of the Micro-Viscometer

**Figure 1:**
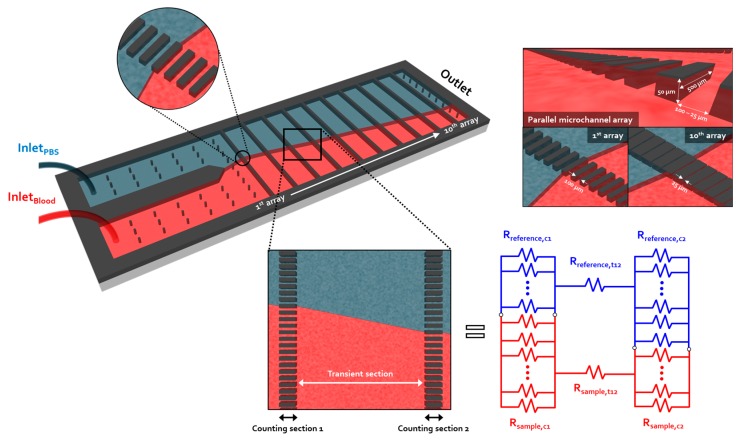
Schematic of the micro-viscometer. The micro-viscometer has a sequence of micro-channel arrays that generate 10 sets of shear rate. The viscosity is derived from the input flow rates for the reference sample fluids and the number of channels filled with both fluids. By varying the channel width at each array, different viscosity values can be obtained. For each array, 100 micro-channels are arranged in parallel. It is designed accordingly to change the width of the channel from the first array to the tenth array to enable each array to form a different shear rate from a single flow condition.

As shown in Figure 1, the viscometer proposed in this study consists of 10 microchannel arrays connected in series at regular intervals. Each array has 100 identical micro-channels. Instead of a radial-shaped device configuration, as in our previously reported viscometer [26], a straight shape was selected since it can reduce the volume of the sample required for the viscosity measurements. In the proposed viscometer, the microchannel array where the viscosity measurement is performed is defined as a counting section. An interval connecting two adjacent arrays is defined as a transient section. Considering that the shear rate is a function of the channel width, each counting section has a different channel width for generating various shear rates at a given flow rate condition (Figure 1). Meanwhile, the transient section has a specific distance between two adjacent counting sections for independently implementing the viscosity measurements.

The approach to measuring the viscosity in the counting section is the same as that described for our previously developed viscometer [26]. In Figure 1, the counting and transient sections filled with reference and sample fluids can be expressed as an electrical circuitry through lumped parameter modeling. The hydraulic resistance of the counting section filled with the two fluids is as follows:

In the micro-viscometer, a micro-channel array forming a different shear rate is defined as a counting section, and a transient section is defined as a section between two adjacent counting sections. According to the lumped parameter modeling, the counting and transient sections where each fluid flows together can be expressed as the hydrodynamic circuitry with hydraulic resistances. Since the hydraulic resistance is a function of geometrical factors and viscosity, the viscosity of the sample fluid can be calculated by comparing the two hydraulic resistances:

(1) $$R_{r}=\frac{12\cdot \mu_{r}\cdot L}{wh^{3}N_{r}}\cdot {\left(1-\frac{192}{\pi^{5}w}\overset{\infty }{\underset{n=1,3,5,\cdots }{\sum }}\tanh\left(\frac{n\pi w}{2h}\right)\right)}^{-1}$$

(2) $$R_{s}=\frac{12\cdot \mu_{s}\cdot L}{wh^{3}N_{s}}\cdot {\left(1-\frac{192}{\pi^{5}w}\overset{\infty }{\underset{n=1,3,5,\cdots }{\sum }}\tanh\left(\frac{n\pi w}{2h}\right)\right)}^{-1}$$

where subscripts *r* and *s* denote the respective reference and sample fluids. In addition, *R*, *w*, *h*, *L*, and *μ*, *N* respectively represent the hydraulic resistance, channel width, height, length, viscosity, and number of channels filled with the fluid. Since the pressure drops from the point where both fluids begin to pass through to the outlet are the same, Poiseuille flow equations for each fluid are described as:

(3) $$\frac{∆P_{s}}{∆P_{r}}=\frac{Q_{s}}{Q_{r}}\cdot \frac{R_{s}}{R_{r}}$$

where Δ*P* and *Q* denote the pressure gradient and input flow rate. Since the two fluids flow in the same geometry of the micro-viscometer, the geometrical factors are the same. Then, Equations (1) and (2) can be combined to obtain a simplified version of the equation:

(4) $$\frac{∆P_{s}}{∆P_{r}}=\frac{Q_{s}}{Q_{r}}\cdot \frac{\mu_{s}}{\mu_{r}}\cdot \frac{N_{r}}{N_{s}}$$

Here, *N_r_* and *N_s_* are the number of channels respectively filled with the reference and sample fluids in the counting section. Thus, because the viscosity of the reference fluid (*μ_r_*) is already a known value, it is possible to predict the viscosity of the sample fluid (*μ_s_*) by determining the input flow rates of each fluid and the number of fluid-filled channels.

(5) $$\mu_{s}=\mu_{r}\cdot \frac{Q_{r}}{Q_{s}}\cdot \frac{N_{s}}{N_{r}}$$

Meanwhile, the micro-viscometer has 10 counting sections with different channel widths because the shear rate ($\dot{\gamma }$) is a function of the channel width, as described in Equation (6) [26].

(6) $$\dot{\gamma }=\frac{6Q_{s}}{wh^{2}N_{s}}\left(\frac{1-\frac{16h}{\pi^{3}w}\sum_{n=1,3,5,\cdots }^{\infty }\frac{1}{n^{3}}\tanh\left(\frac{n\pi w}{2h}\right)}{1-\frac{192h}{\pi^{5}w}\sum_{n=1,3,5,\cdots }^{\infty }\frac{1}{n^{5}}\tanh\left(\frac{n\pi w}{2h}\right)}\right)$$

Instead of changing the input flow rates, as in previous viscometers, the proposed micro-viscometer has a varying channel width in each counting section, which generates multiple shear rate conditions from a given flow rate.

### 2.5. Determination of the Optimal Flow Rate Ratio

For accurate viscosity measurements, the input flow rates of the reference and sample fluids should be considered because the flow rate ratio determines the number of channels for two fluids. In an ideal situation, the number of channels does not affect the measurement accuracy. In reality, however, a channel may be mistakenly counted due to a practical issue, such as fluidic fluctuation. In particular, if the number of channels for the reference fluid (*N_r_*), a denominator in Equation (5), is too small, then a change in a single channel with the reference fluid will have a large influence on the viscosity measurement. In other words, as the number of reference fluid channels increases, a reliable viscosity measurement becomes possible. In the presented micro-viscometer, therefore, a higher PBS to blood flow rate ratio is chosen in order to increase the number of channels occupied by a reference fluid (PBS).

**Figure 2:**
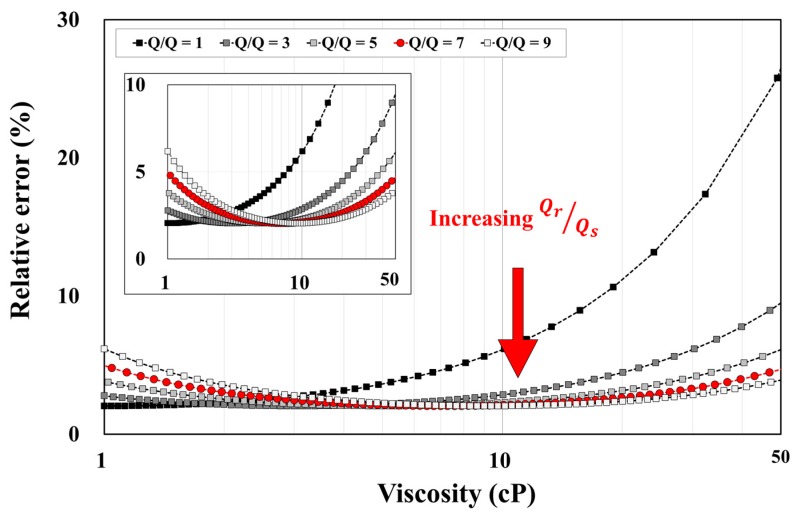
Optimal flow rate ratio of the reference and sample fluids. The x-axis represents the viscosity to be measured in the counting section; the y-axis represents the expected relative error that is calculated for the case when a single channel for PBS is mistakenly counted. Flow rate ratio (*Q*/*Q*) means the ratio of the reference (*Q_r_*) and sample fluid (*Q_s_*). As the input flow rate of the reference fluid increases relative to the flow rate of the sample fluid, the relative error decreases in the viscosity range above 10 cP. However, as depicted in the inset, the increasing flow rate ratio also induces an elevated relative error in the low viscosity range near 1 cP. Consequently, the optimal condition of the flow rate ratio, which ensures a relative error of less than 5% over the whole range of viscosities, is set to seven (red circle).

The graph in Figure 2 shows the theoretical calculation of the relative error for the case when a single PBS channel is mistakenly counted. The flow rate ratio is increased from one to nine. First, when the flow rate ratio is one, the relative error increases with respect to the viscosity to be measured. For the viscosity seven, the maximum relative error is 5% for the viscosity range of 1–50 cP. In this study, the input flow rate of the reference fluid is set to be seven-fold higher than the sample fluid.

## 3. Results and Discussion

### 3.1. Parametric Study for Design Optimization of the Micro-Viscometer

A parametric study was conducted to determine the optimal geometry of the micro-viscometer. The design factor in the study was the hydraulic resistance ratio of the counting and transient sections. Previously, we proved that the underestimation by the Fahraeus effect, which causes a viscosity reduction in the blood flow in a microchannel [27], does not occur when the equivalent cross-sectional area of micro-channels is larger than 400 μm [26]. In the presented micro-viscometer, however, the optimal flow rate ratio is fixed at seven so that the number of channels filled with the blood sample is less than in the case of the flow rate ratio of one. In fact, when the viscosity of a typical blood sample with 45% hematocrit is measured by the micro-viscometer at room temperature, the equivalent cross-sectional area of the counting section is approximately 250–580 μm, and the transient section is 800–910 μm. This implies that the Fahraeus effect occurs in some parts of the counting sections, whereas it apparently does not occur in the transient section. In this study, as an alternative strategy to avoid this issue, proper selection of the hydraulic resistance ratio of the counting and transient sections was investigated for the accurate measurement of the blood viscosity.

Prior to the parametric study, the channel widths of the counting sections were fixed in the range of 25 to 100 μm for enabling various shear rate conditions. The height of the channel was equal to 50 μm throughout the device. Thus, the geometrical variable to be modulated in the parametric study was the length of the counting and transient sections. The specifications of the micro-viscometer in this study are summarized in Table 2. The lengths of the counting and transient sections were set as parameters, and the blood sample viscosity was measured by changing the channel length.

**Figure 3:**
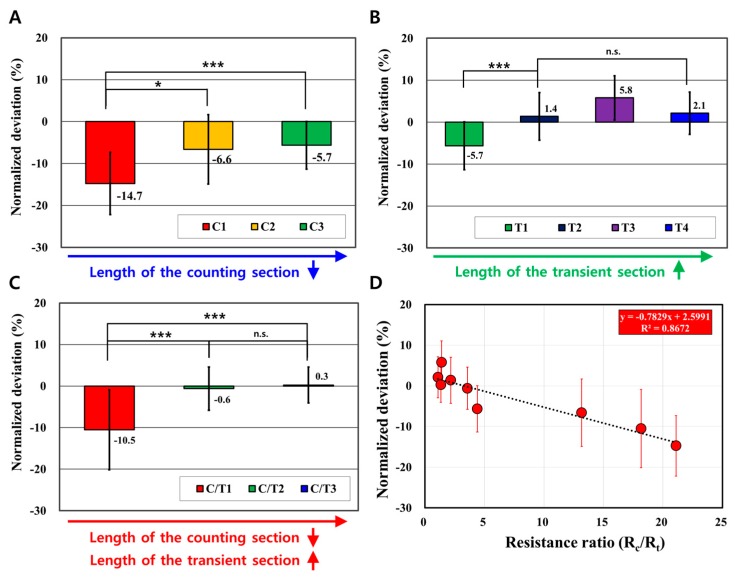
Parametric study for the design optimization of the micro-viscometer. (**A**) At a given length of the transient section (1000 μm), it is confirmed that, as the length of the counting section is decreased (from C1 to C3), the relative error significantly decreases. (* p < 0.05, *** p < 0.001) Specifically, the underestimation seems to be alleviated because the hydraulic resistance of the counting section, which is related to the Fahraeus effect, is relatively decreased. (**B**) At a given counting section length (500 μm), it is confirmed that the relative error significantly decreases as the length of the transient section increases (from T1 to T4). This is also caused by the counting section, which has a relatively low value of hydraulic resistance. (**C**) When the length of the counting section is reduced and the length of the transient section is simultaneously increased (from C/T1 to C/T3), the underestimation decreases, and eventually the relative error converges. (**D**) The resistance ratio between the counting and transient sections is expressed as *R_c_*/*R_t_*, represented as the x-axis in the graph. The underestimation due to the Fahraeus effect occurs as the counting section resistance increases. On the contrary, it is confirmed that an accurate viscosity measurement is possible when the resistance ratio converges to be close to zero (y-intersect: 2.5991%).

Initially, the length of the counting section was reduced from 2500 μm to 500 μm (from C1 to C3), while the length of the transient section was fixed at 1000 μm. The results of the viscosity measurements are given in Figure 3A, where it is shown that the underestimation is gradually decreased. This implies that the blood viscosity was less underestimated by the lower hydraulic resistance of the counting section. For the case of C3, however, the relative error of −5.7 ± 5.7% remained due to the insufficient length of the transient section.

The second parameter, which is the length of the transient section, varied from 1000 to 4000 μm (from T1 to T4). For this case, the length of the counting section was fixed at 500 μm. As shown in Figure 3B, the underestimation does not occur as the length of the transient section increases. This reflects that the Fahraeus effect is not operative because the hydraulic resistance of the transient section increases when compared to the counting section.

For the third parametric study, the length of the counting section was reduced from 2000 to 500 μm, while the length of the transient section was simultaneously increased from 2000 to 4000 μm (from C/T1 to C/T3). As a result, underestimation in the micro-viscometer (C/T1) with a long counting section still occurred, as shown in Figure 3C. However, as the length of the counting section gradually decreased and the length of the transient section increased, the relative error reduced to a very low level. (C/T2: −0.6 ± 5.2%, C/T3: 0.3 ± 4.3%). Once the hydraulic resistance ratio between counting and transient sections increased, the severe underestimation of the viscosity measurements occurred. In contrast, the decreasing resistance ratio showed accurate viscosity measurements. Consequently, the design of the micro-viscometer selected in this study was C/T3. It may provide an experimental error of 0.56–2.36%, as per regression analysis in Figure 3D. Consequently, it is noted that the Fahraeus effect could be neglected in the micro-viscometer via optimization of the hydraulic resistance ratio between the counting and transient sections.

### 3.2. Preliminary Test: Newtonian Viscosity

To demonstrate the concept of the hydrodynamic spreading in the micro-viscometer, the viscosity of the Newtonian fluid was measured. For visualization, 1% (*v*/*v*) fluorescent particles (0.3 μm in diameter) were mixed with DI water, which is a reference fluid. As shown in Figure S1, the viscosity of the 8% SDS solution obtained by the micro-viscometer was about 1.8 ± 0.1 cP. It showed a Newtonian behavior, i.e. the viscosity was independent of the shear rate. Furthermore, it showed good agreement with the one obtained from the rotational viscometer (4.3% in relative error).

### 3.3. Numerical Demonstration

**Figure 4:**
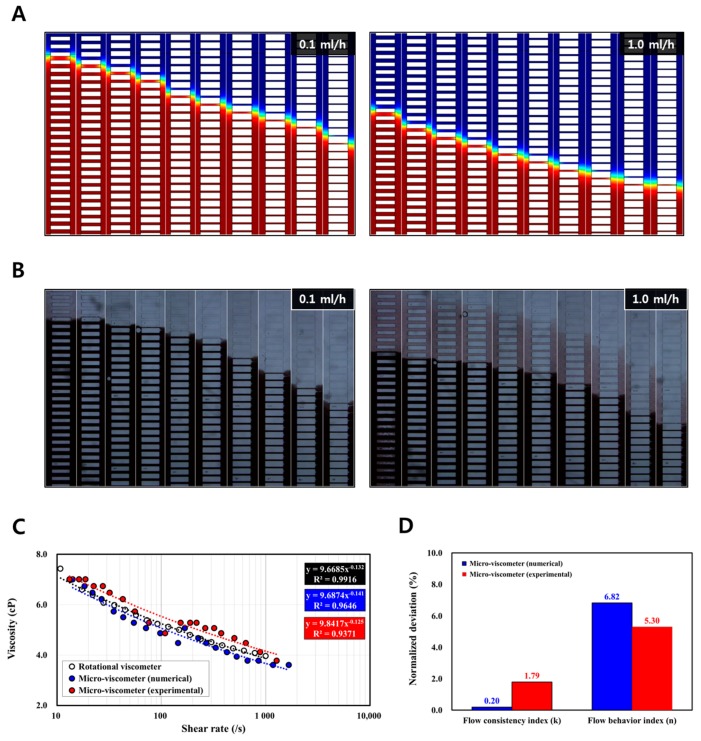
Numerical and experimental demonstrations of the micro-viscometer. (**A**) For the given flow rate conditions (0.1 and 1.0 mL/h), the captured images of 10 counting sections showing the fluidic boundaries are depicted. The number of channels filled with the sample fluid (red) range from a maximum of 50 to a minimum of 39 in the low shear rate regime (14.2–145.4/s), and to a minimum of 34 in the high shear rate regime (168.8–1668.3/s). (**B**) Captured images showing fluidic boundaries in the 10 counting sections. The number of channels filled with the blood sample decreased from a maximum of 50 to a minimum of 41 (for the flow rate of 0.1 mL/h), and decreased from a maximum of 43 to a minimum of 35 (for the flow rate of 1.0 mL/h). (**C**) The viscosity values from the numerical study (blue circle) are obtained from two flow rate conditions, which can be fitted with 9.69 (for k) and −0.14 (for n) using the power-law model. Also, 20 viscosity values (red circle) obtained from the experiment are fitted using the power-law model with 9.84 (for k) and −0.13 (for n). (**D**) It is confirmed that the numerical result shows relative errors of 0.2% (for k) and 6.8% (for n) and the experimental result shows relative errors of 1.79% (for k) and 5.30% (for n). This implies that the micro-viscometer design concept enables accurate measurement of the shear-thinning viscosity.

Figure 4A depicts the obtained images showing the change in the fluidic boundary at 10 counting sections from the numerical simulation. Blue and red represent the respective reference and sample fluids. Ten data points from the low shear rate regime (14.2–145.4/s), and another 10 data points from the high shear rate regime (168.8–1668.3/s), were obtained from the two sets of input flow rates (0.1 and 1.0 mL/h). The number of channels for the sample fluid decreased from 50 to 34, which showed the shear-thinning behavior of the non-Newtonian fluid. The results in Figure 4C demonstrate the relative errors of 0.2% (for k) and 6.82% (for n) compared with the results obtained from the rotational viscometer. From these numerical analyses, it was conceptually verified that the viscometer with the proposed design operated accurately.

### 3.4. Experimental Demonstration 

Figure 4B depicts the captured microscopic images with the change in the fluidic boundary obtained from the flow rates of 0.1 and 1.0 mL/h. Black denotes the red blood cell suspension in PBS; the transparent part represents the PBS. From the experiment, 20 sets of viscosity values in the shear rate of 13.2–1277.5/s were obtained (Figure 4C). The number of channels for PBS increased from 50 to 65, whereas the number of blood channels decreased from 50 to 35. Similar to the result demonstrated by the numerical study, the experimental result showed shear-thinning behavior with relative errors of 1.79% (for k) and 5.30% (for n) when compared to the reference data from the rotational viscometer. (Figure 4D).

**Figure 5:**
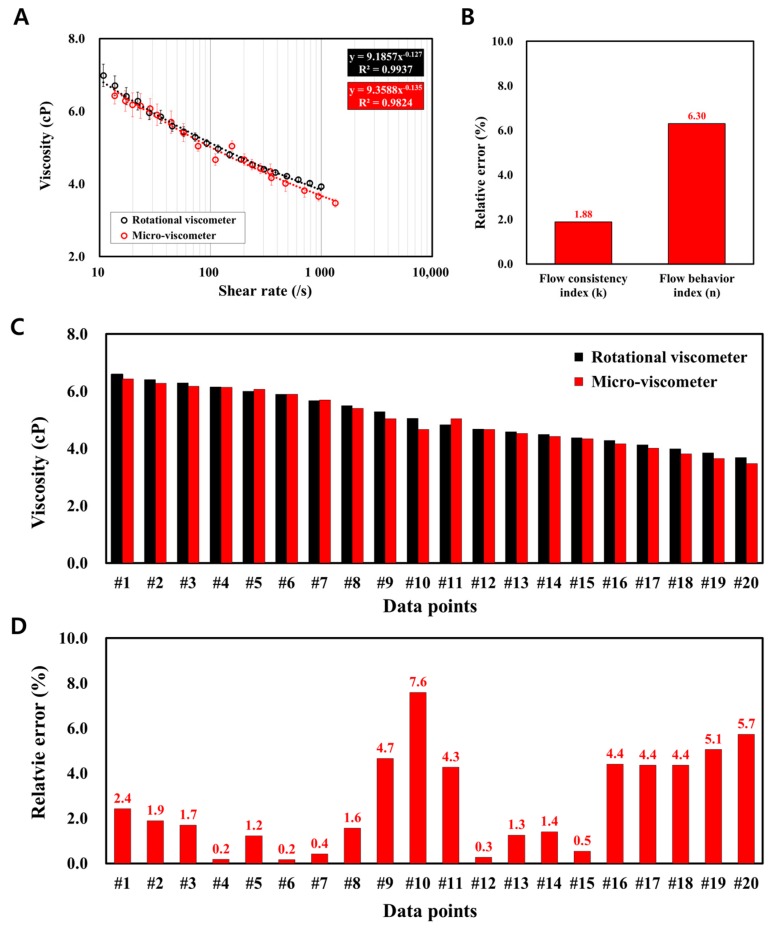
Experimental reliability test of the micro-viscometer. (**A**) Repeated viscosity measurements were performed (N = 7) with the proposed micro-viscometer. Each measurement provides very similar results (averaged standard deviation of 4.25% for seven sets of viscosity measurements). The result also shows a very similar shear-thinning viscosity of the blood compared to the result from the rotational viscometer. (**B**) Relative errors for the two indices (k and n) are 1.88% and 6.30%, respectively. (**C**,**D**) According to the *t*-test, there are no statistically significant differences (*p* > 0.05) for 14 data points, and the averaged relative error is 1.56%. Although the remaining six data points show a significant difference (*p* < 0.05), the averaged relative error is 5.35%. This implies that the micro-viscometer shows reliability in repeated viscosity measurements.

Furthermore, the accuracy of the micro-viscometer in the viscosity measurements was evaluated by repeatedly measuring the blood viscosity (*N* = 7). The viscosity measurements of the blood sample were performed with both the micro-viscometer and rotational viscometer, and they were compared as depicted in Figure 5A. In terms of the flow indices, the relative errors of k and n were 1.9 and 6.3%, respectively (Figure 5B). Based on the *t*-test, it was determined that the 20 sets of viscosity values from the micro-viscometer showed statistically significant agreement with the ones from the rotational viscometer (Figure 5C–D). It was found that 14 data points showed no significant difference (*p* > 0.05) and the average relative error was 1.56%. For the remaining six data points, they showed significant differences (*p* < 0.05); however, the relative error was on average equal to 5.35%. Repeated viscosity measurements demonstrated that the micro-viscometer provided reliable performance.

### 3.5. Blood Sample Viscosity Measurements with Variation in Hematocrit Levels

**Figure 6:**
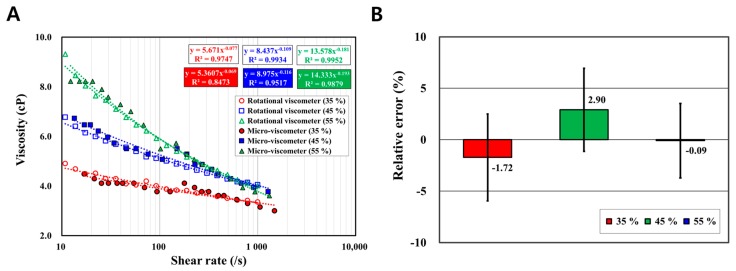
Viscosity measurement with variation in hematocrit levels. (**A**) Viscosity measurements of the three samples with different hematocrit levels (35%, 45%, and 55%) were performed. The results show 5.36, 8.98, and 13.52 (for k) and −0.08, −0.11, and −0.18 (for n), respectively. Thus, an increase in both indices proves that blood samples with higher hematocrit levels have higher viscosities as well as higher magnitudes of shear-thinning behavior. The relative errors are 5.5%, 6.4%, and 5.6% (for k), and 10.4%, 6.4%, and 6.6% (for n), respectively. (**B**) A comparison of the 20 data points obtained from each sample show average relative errors of −1.72 ± 4.21%, −2.90 ± 4.03%, and −0.09 ± 3.61%, respectively. Consequently, it is demonstrated that the micro-viscometer recognizes viscosity changes due to the variation in hematocrit levels.

Viscosity measurements were performed on three blood samples with variation in hematocrit levels by the micro-viscometer. Hematocrit, which is the volume of red blood cells corresponding to the volume of the total sample volume, is considered an important factor affecting blood viscosity. Thus, the three blood samples were prepared with hematocrit levels of 35%, 45%, and 55%. The hematocrit level of each sample was checked by using a micro-centrifuge (HA-200, Hanil Science Medical, Daejeon, Korea). The experimental results with the three samples showed 5.671, 8.437, and 13.578 in k and −0.077, −0.109, and −0.181 in n, respectively. As shown in Figure 6A, it was confirmed that the viscosity of the blood sample increased as the hematocrit level increased. The relative errors for the three samples compared to the reference data were 5.5%, 6.4%, and 5.6% (for k) and 10.4%, 6.4%, and 6.6% (for n). Additionally, the average relative errors were 1.72 ± 4.21%, −2.90 ± 4.03%, and −0.09 ± 3.61%, respectively (Figure 6B). This implies that the micro-viscometer recognized the blood viscosity changed by the hematocrit levels.

## 4. Conclusions

The micro-viscometer developed in this study enables the measurement of 10 sets of blood viscosity over a wide range of shear rates from only a single flow rate. To ensure accurate viscosity measurements, the counting section and transient section in the micro-viscometer were optimized through a parametric study. The micro-viscometer measurement performance was verified by numerical and experimental analyses, showing a relative error of 6.8% (numerical) and 5.3% (experimental). Repeatability test results demonstrated good reproducibility with an average relative error of 2.69 ± 2.19%. It was also experimentally confirmed that the micro-viscometer accurately measured the viscosity of blood samples that have different hematocrit levels. In conclusion, it was proven that the proposed micro-viscometer can acquire 10-fold more data points of the blood viscosity than previous pressure-driven viscometers for the same flow rate condition. Furthermore, it guaranteed reliable viscosity measurements by repeated demonstrations.

## Figures and Tables

**Table 1:** Numerical conditions of the reference and sample fluids.

Property	Density [kg/m^3^]	Viscosity [cP]	Mass Flow Rate [kg/s]	
			Flow Rate 1	Flow Rate 2
**Reference fluid**	1000	0.001	1.95 × 10^−7^	1.95 × 10^−6^
**Sample fluid**	1025	k = 9.6685 n = −0.132	2.78 × 10^−^^8^	2.78 × 10^−7^

**Table 2:** Micro-viscometer specifications for the parametric study.

Type	Counting Section	Transient Section
C1	2500	1000
C2	1500	
C3	500	
T1	500	1000
T2		2000
T3		3000
T4		4000
C/T1	2000	2000
C/T2	1000	3000
C/T3	500	4000

## Supplementary Materials

The following are available online at http://www.mdpi.com/1424-8220/17/6/1442/s1, Figure S1: Experimental demonstration of the micro-viscometer using a Newtonian fluid. Viscosity of the 8% SDS solution is obtained by the micro-viscometer (red circle) and the rotational viscometer (white circle). For the rotational viscometer, the averaged viscosity of 20 data points under 17.3–1000/s in shear rate is 1.88 cP. For the micro-viscometer, the averaged viscosity under 21.9–1505.9/s in shear rate is 1.78 cP. It shows good agreement compared to the result from the rotational viscometer (4.3% in relative error). In addition, it is demonstrated that the proposed micro-viscometer can measure the shear-independent viscosity of the Newtonian fluid.
