# Supplementary material for: Micro-Viscometer for Measuring Shear-Varying Blood Viscosity over a Wide-Ranging Shear Rate
Source: Sensors (Basel). 2017 Jun 20;17(6):1442. doi: 10.3390/s17061442 (PMC5492087; doi:10.3390/s17061442)
Supplement: Supplementary file 1 [file sensors-17-01442-s001.pdf]

sensors

MDPI

Electronic Supplementary Material

Micro-Viscometer for Measuring Shear-Varying Blood Viscosity over a Wide-Ranging Shear Rate

Byung Jun Kim [1], Seung Yeob Lee [1], Solkeun Jee [2], Arslan Atajanov [2] and Sung Yang [1,2,*]

[1] Department of Biomedical Science and Engineering, Gwangju Institute of Science and Technology, Gwangju 61005, Korea; gene392@gist.ac.kr (B.J.K.); syl@gist.ac.kr (S.Y.L.)

[2] School of Mechanical Engineering, Gwangju Institute of Science and Technology, Gwangju 61005, Korea; sjee@gist.ac.kr (S.J.); arslanrcx@gist.ac.kr (A.A.)

* Correspondence: syang@gist.ac.kr; Tel.: +82-62-715-2407; Fax: +82-62-715-2384

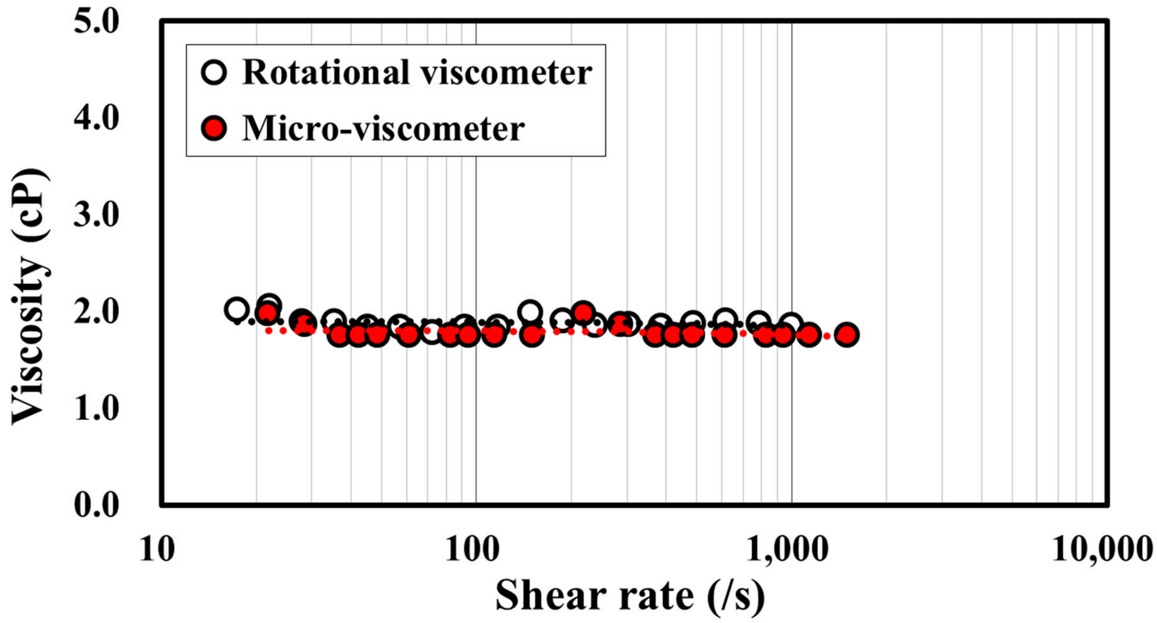

Figure S1. Experimental demonstration of the micro-viscometer using a Newtonian fluid. Viscosity of the 8% SDS solution is obtained by the micro-viscometer (red circle) and the rotational viscometer (white circle). For the rotational viscometer, averaged viscosity of 20 data points under 17.3–1000/s in shear rate is 1.88 cP. For the micro-viscometer, averaged viscosity under 21.9–1505.9/s in shear rate is 1.78 cP. It shows a good agreement compared to the result from the rotational viscometer (4.3% in relative error). And it is demonstrated that the proposed micro-viscometer can measure the shear-independent viscosity of the Newtonian fluid.
